# Copper-Assisted Direct Growth of Vertical Graphene Nanosheets on Glass Substrates by Low-Temperature Plasma-Enhanced Chemical Vapour Deposition Process

**DOI:** 10.1186/s11671-015-1019-8

**Published:** 2015-08-04

**Authors:** Yifei Ma, Haegyu Jang, Sun Jung Kim, Changhyun Pang, Heeyeop Chae

**Affiliations:** School of Chemical Engineering, Sungkyunkwan University (SKKU), Suwon, 440-746 Republic of Korea; SKKU Advanced Institute of Nanotechnology (SAINT), Sungkyunkwan University (SKKU), Suwon, 440-746 Republic of Korea

**Keywords:** Low temperature, PECVD, Vertical graphene nanosheets, Glass substrate, Copper-assisted growth

## Abstract

Vertical graphene (VG) nanosheets are directly grown below 500 °C on glass substrates by a one-step copper-assisted plasma-enhanced chemical vapour deposition (PECVD) process. A piece of copper foil is located around a glass substrate as a catalyst in the process. The effect of the copper catalyst on the vertical graphene is evaluated in terms of film morphology, growth rate, carbon density in the plasma and film resistance. The growth rate of the vertical graphene is enhanced by a factor of 5.6 with the copper catalyst with denser vertical graphene. The analysis of optical emission spectra suggests that the carbon radical density is increased with the copper catalyst. Highly conductive VG films having 800 Ω/□ are grown on glass substrates with Cu catalyst at a relatively low temperature.

**Electronic supplementary material:**

The online version of this article (doi:10.1186/s11671-015-1019-8) contains supplementary material, which is available to authorized users.

## Background

Various carbon nanostructures have received enormous attention recently due to their excellent physical properties. For example, graphene, an *sp*^*2*^-hybridized two-dimensional carbon material, shows excellent physical properties in intrinsic mobility [1, 2], mechanical strength [3], optical transmittance [4] and electric conductivity [5]. These properties make graphene a promising material in a wide range of applications of electronics [6, 7], optoelectronics [8, 9], sensors [10, 11], batteries [12] and supercapacitors [13–17]. Enormous processes have been developed for the synthesis of graphene including exfoliation from highly oriented pyrolytic graphite (HOPG), reduction of chemically exfoliated graphene oxide (RGO), thermal decomposition of SiC and chemical vapour deposition (CVD). In general, these processes generate in-plane-oriented monolayer or multi-layer graphene films.

Recently, three-dimensional (3D) graphene attracts attention due to its high surface-area-to-volume ratio [1]. Vertical graphene (VG) nanosheet is one of the popular 3D carbon structure materials [10], which has been applied to various applications of field emitters [7, 18], supercapacitors [19–22] and batteries [23, 24]. In practice, VG films have been typically grown on metal substrates at a relatively high temperature [10, 16], close to 1000 °C, which limits the use of various low-melting-temperature substrates. Yang et al. reported the growth of VG films on dielectric substrates (SiO_2_) [25] at the temperature of 900 °C. However, the growth rate drops significantly at the temperature below 900 °C. Liu et al. reported the synthesis of carbon nanosheets on a metal-coated glass at a low temperature. In fact, it is a kind of process to grow carbon-based material directly on metal [26]. Recently, the catalytic effect of copper on graphene growth has been reported in high-temperature CVD processes [27, 28], whereas it has not been studied in a plasma-enhanced chemical vapour deposition (PECVD) process. Furthermore, low operation temperature is necessary for an economic and facile process, which can be more feasible for industrial application. Especially, it can pave the way for more applicable substrate materials [29]. For instance, glass is a widely used commercial material with a cheap price but low melting temperature, which should be adopted in a low-temperature process.

In this work, VG films were grown on glass substrates in plasma-enhanced CVD with copper foils at a relatively low temperature, and the properties of vertical graphene films were investigated with the catalytic effect of copper foil in the PECVD process.

## Methods

**Fig. 1:**
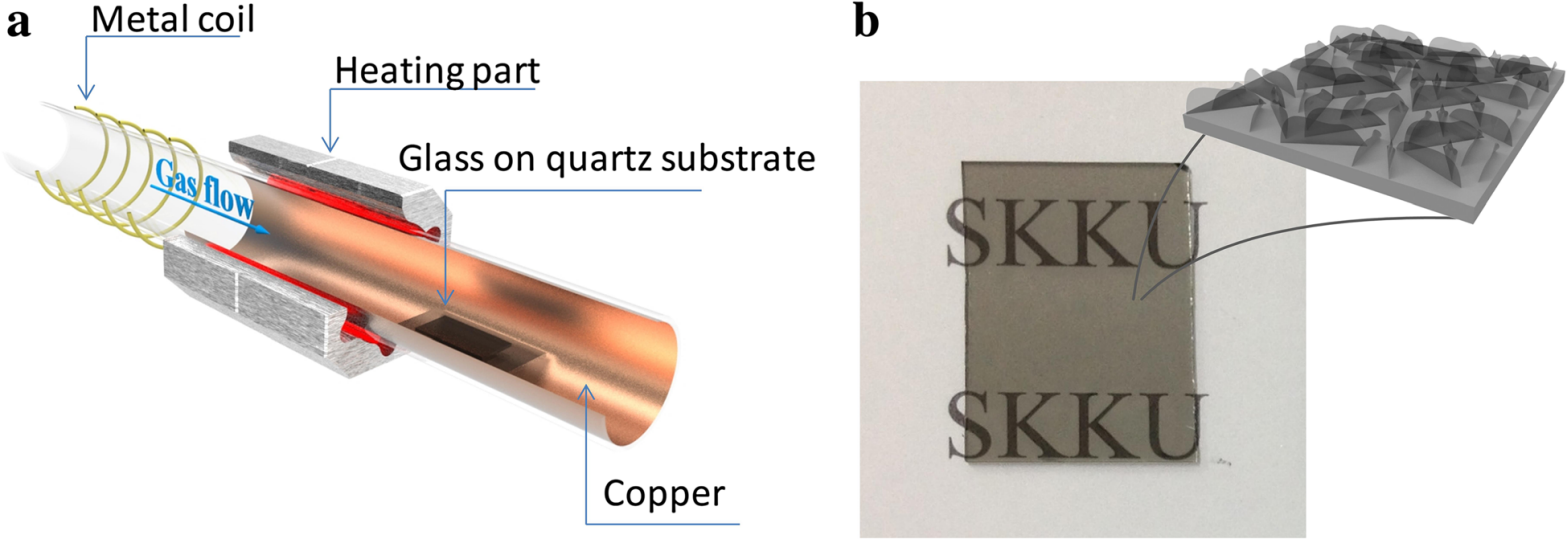
Schematic diagram of the reactor and a photo of the vertical graphene (VG) film. Schematic diagram of the plasma-enhanced chemical vapour deposition (PECVD) reactor (**a**), and a photo of the VG film deposited on a glass substrate (**b**)

Vertical graphene nanosheets were grown in a radio-frequency (RF) inductively coupled plasma (ICP) reactor as shown in Fig. 1. A piece of copper foil (50 × 25 cm^2^) was located inside of the quartz tube reactor. The temperature in the centre of the heating area can be increased up to 900 °C by the lamp heater located in the centre of the reactor. A pre-cleaned glass substrate was placed outside (downstream zone) of the direct heating zone, and the temperature around the glass substrates was maintained at about 500 °C to prevent glass deformation. Before the growth of the graphene nanosheets, the glass substrate was cleaned by H_2_ for 2 min with 100 W of plasma power. After the cleaning, 2 sccm of C_2_H_2_ and 1 sccm of H_2_ were introduced into the tube reactor and 280 W of plasma power was supplied to the coils outside the reactor. The processing pressure was kept at 12 mTorr throughout the growth process. The growth rate was calculated by dividing the height by the growth time from the scanning electron microscopy (SEM) images. After growing graphene nanosheets on the glass substrate, the system was cooled down to room temperature slowly. A uniform VG film obtained on a glass substrate is shown in Fig. 1b.

Optical emission spectra (OES) were taken during the growth process by a high-resolution spectrometer (HR4000CG-UV-NIR, Ocean Optics). The VG film structure was analysed with field emission SEM (JEOL, JSM7401F). High-resolution transmission electron microscopy (HRTEM, JEM-2100F JEOL) was taken to confirm successful growth of graphene in nanoscale. Chemical elements of as-prepared films were determined by an energy-dispersive spectrometer (EDS, JEOL, JSM 6700F). Carbon bonding structure was analysed by Raman spectroscopy (Renishaw, RM-1000 Invia) with a wavelength of 532 nm (Ar^+^ ion laser). The optical transmittance of VG films was determined by a UV-vis spectrophotometer (UV-650, JASCO) in the visible and infrared ranges.

## Results and Discussion

**Fig. 2:**
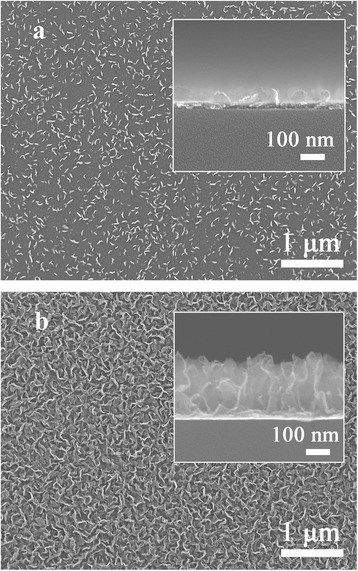
SEM of VG on glass substrate without and with copper catalyst. SEM images of the VG grown on a glass substrate for 20 min without copper catalyst (**a**) and for 12 min with copper catalyst (**b**). The *inset images* are the cross-sectional SEM images of the samples

In the process of growing carbon materials by CVD, such as carbon nanotubes and graphene, metal foils (Cu, Ni, Co, etc.) are typically adopted as substrates [7, 20]. However, during the growth process of the VG film by PECVD, we found that the VG films can be also grown on dielectric substrates (e.g. glass) even at temperature as low as 500 °C without any metal substrate, whereas the growth rate is quite low under the low growth temperature. Thus, in order to grow dense VG with a high growth rate, copper was adopted as a catalyst of the low-temperature PECVD process. The significant growth enhancement effect of copper is clearly shown in the SEM images in Fig. 2. Without the copper catalyst, a 100-nm-thick VG nanosheet film is grown in 20 min on a glass substrate (Fig. 2a inset), with a growth rate of 5 nm/min. In this condition, the small VG nanosheets are sparsely spread on the substrate as shown in Fig. 2a. In contrast, a 270-nm-thick VG film is obtained in 12 min with the copper foil inside the reactor. The growth rate is significantly increased to 28 nm/min, and the VG size is enlarged in the copper-assisted PECVD process.

**Fig. 3:**
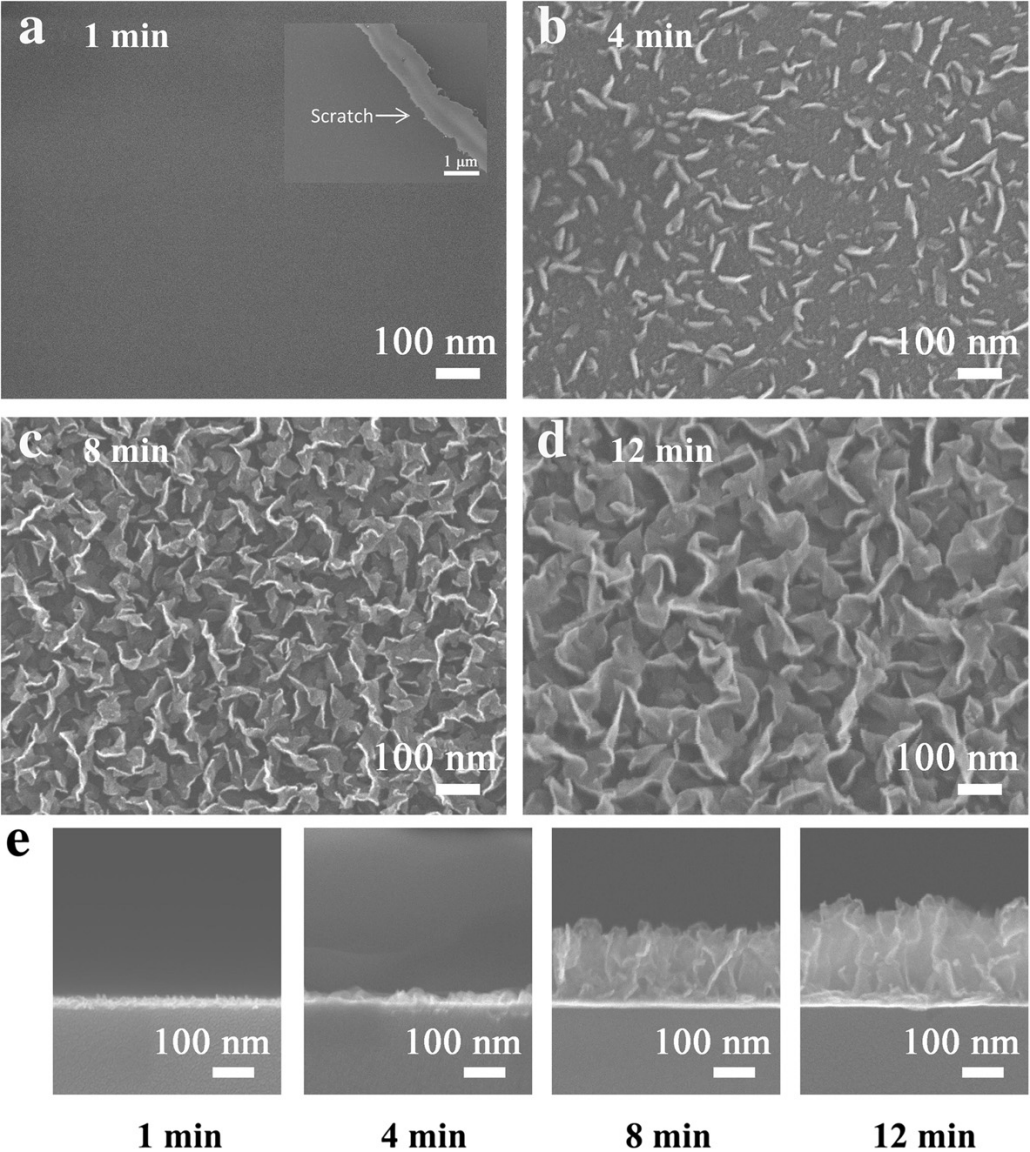
SEM images of growth time effect on VG. SEM images of the VG grown for 1 min (**a**), 4 min (**b**), 8 min (**c**) and 12 min (**d**). The *inset image* in (**a**) shows a scratch made by tweezers. **e** Cross-sectional SEM images of the VG grown on glass with growth times of 1, 4, 8 and 12 min, respectively

Several VG films are reported on various substrates of Ni, Si, SiO_2_ and Cu by PECVD at high temperature [1, 7, 30]. However, the growth mechanism of VG films has not been cleared on glass substrates with copper catalysts. The evolution of VG films on glass substrates is monitored by varying the growth time from 1 to 12 min as shown in Fig. 3. A thin layer is grown on the glass surface within the first 1 min, but no obvious VG nanosheets can be found. The thin layer plays a role as a buffer layer [31], connecting the substrate and the VG nanosheets. The existence of the buffer layer is confirmed by a scratch made by tweezers as shown in the inset. As the growth time is increased to 4 min, a large amount of VG nanosheets appear on the buffer layer (Fig. 3b). As the growth time is increased to 8 and 12 min, the VG nanosheets are further grown and connected densely. The height of the VG nanosheets increases continuously with the growth time (Fig. 3e).

**Fig. 4:**
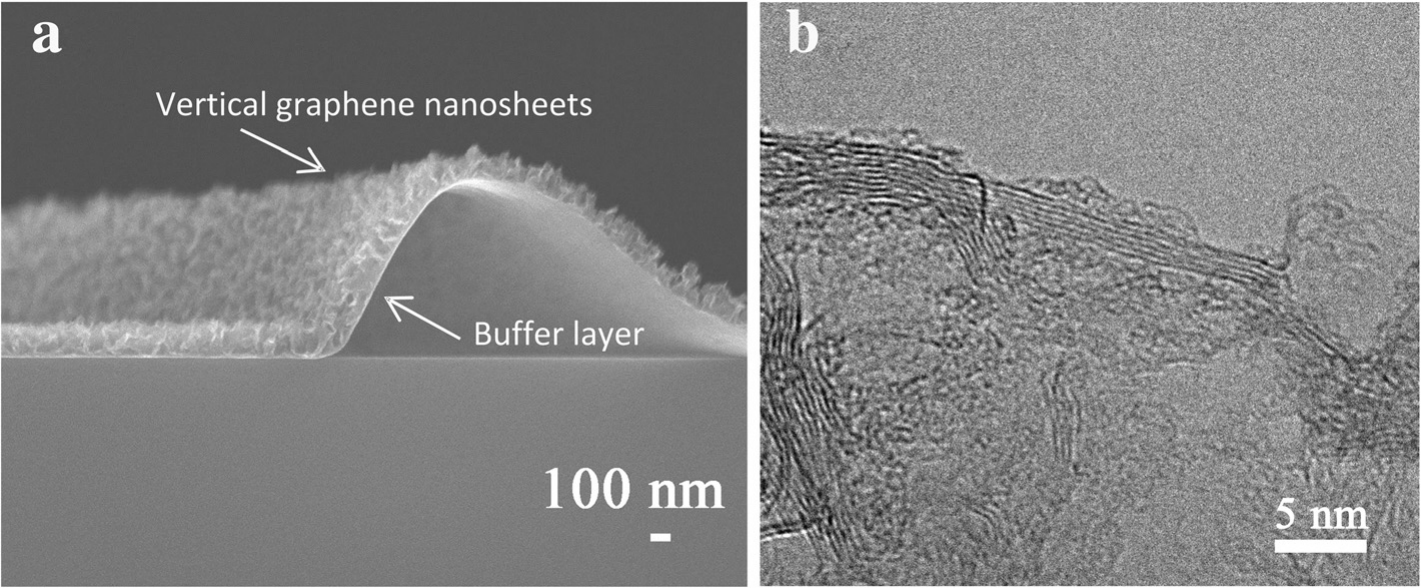
SEM and TEM images of a VG film. **a** Cross-sectional SEM image of a crispate VG film. **b** TEM image of a VG nanosheet structure on the VG film. The VG film was grown for 8 min

The vertical structure of the crispate VG film was investigated by the cross-sectional SEM analysis as shown in Fig. 4a. The VG film on the glass substrate is composed of two layers of a horizontal buffer layer and VG nanosheets on that buffer layer. The buffer layer is believed to reduce the mismatch of the atomic structure between the glass and VG and similar results observed elsewhere [32], and the VG nanosheets are grown on the buffer layer with less stress. Multi-layer graphene structure in the VG nanosheets was identified by TEM analysis show as shown in Fig. 4b.

**Fig. 5:**
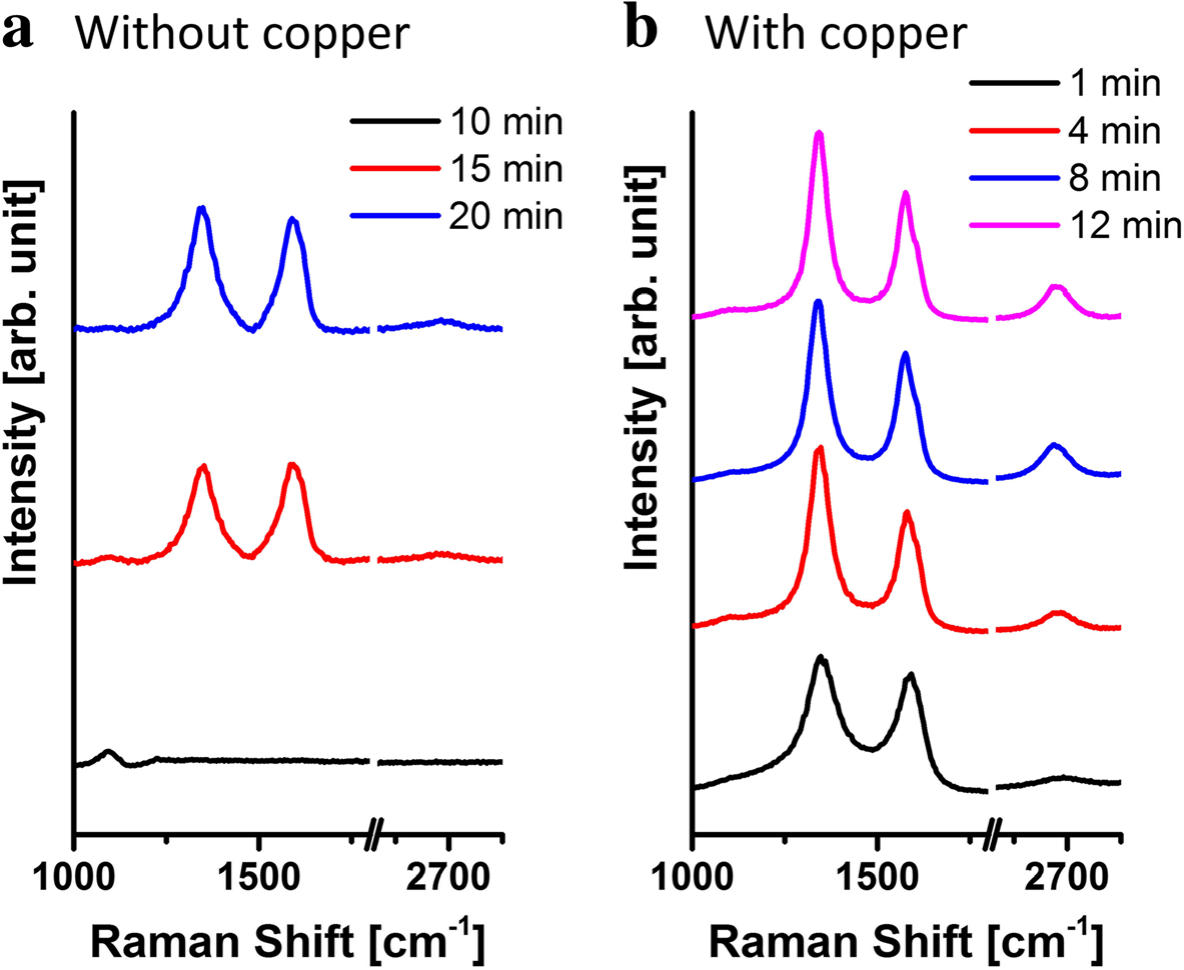
Raman spectra of copper catalytic effect. Raman spectra of VG nanosheets grown without (**a**) and with (**b**) assistance of a copper catalyst for different growth times

Raman spectra of the VG films were taken with the different growth time to evaluate the carbon bonding structure as shown in Fig. 5. The VG grown without copper catalyst does not show the characteristic D and G peaks in the Raman spectra within the growth time of 10 min, indicating the deposition of the base layer of amorphous carbon. Until 20 min of the growth time, a carbon layer is deposited and a barely visible (2D) peak can be found at 2670 cm^−1^. The growth rate is significantly enhanced when the copper catalyst is applied in the PECVD process as shown in Fig. 5b. Significant D and G peaks are observed in the spectrum of the VG film even with a growth processing time of 1 min. However, the low 2D peak signal intensity at 2670 cm^−1^ implies an amorphous carbon structure [33]. This layer of amorphous carbon is believed to serve as a buffer layer as mentioned earlier. The signal intensity of the 2D peak starts to increase with the increase of the growth time indicating the growth of VG nanosheets on substrate.

**Fig. 6:**
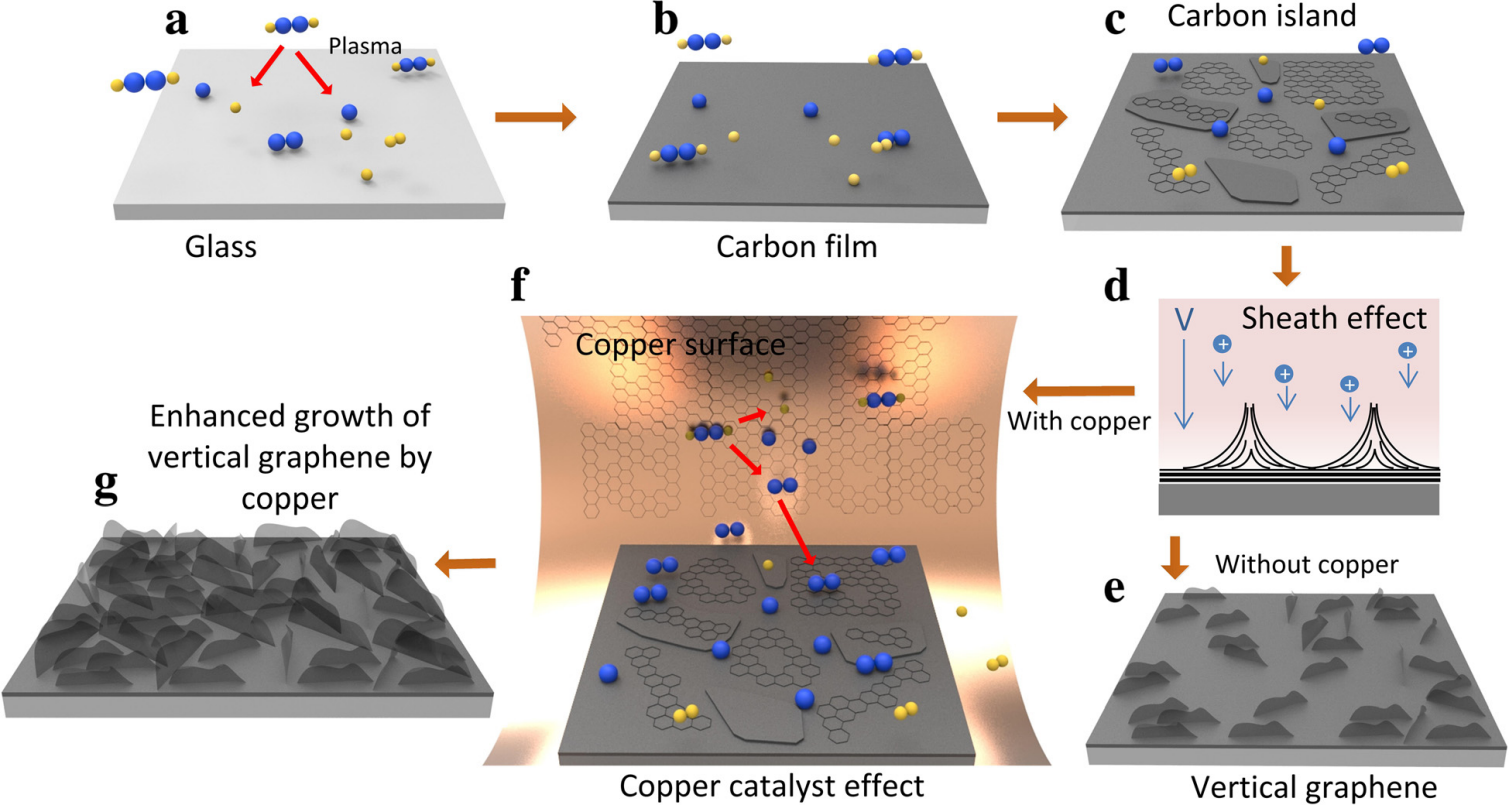
Schematic growth process of VG film on glass substrate in a PECVD system. **a** Dissociation of carbon-hydrogen bonds by plasma. **b** Formation of the carbon buffer layer on the glass substrate. **c** Simultaneous growth of graphene and carbon islands. **d** Sheath effect and ion bombardment between bulk plasma and the substrate. **e** Sparse distribution of VG nanosheets prepared by PECVD process without the copper catalyst on the glass substrate. **f**, **g** Schematic growth process of the VG film by enhancement of copper catalyst. **f** Dissociation of hydrocarbon gas on the surface of copper. The dissociated reactive radicals transport to bulk plasma and increase the radical density. **g** Dense distribution of the VG nanosheets prepared by PECVD process with the copper catalyst on the glass substrate

**Fig. 7:**
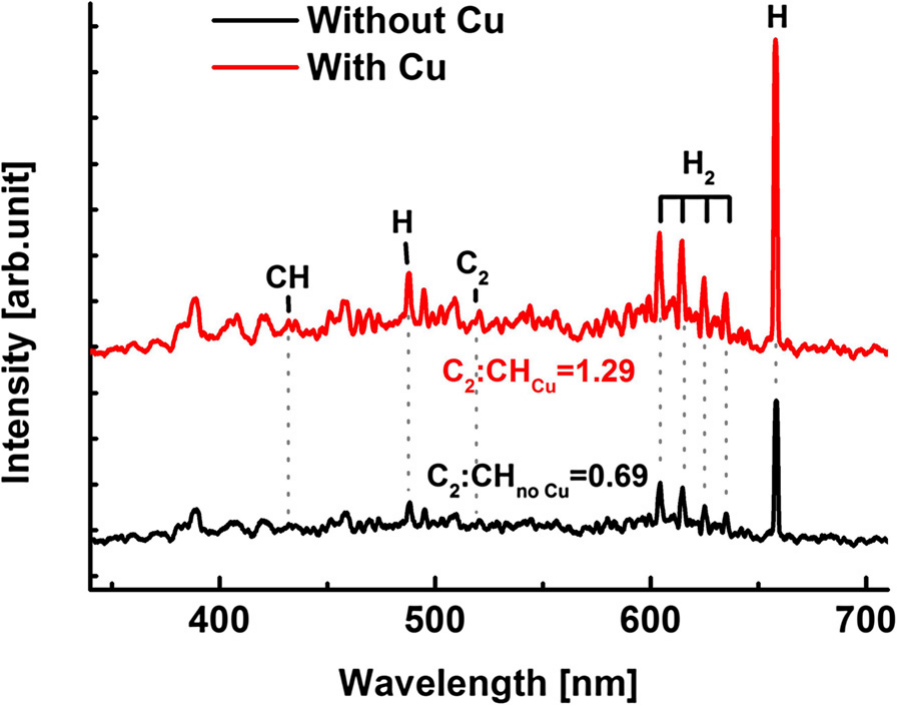
Optical emission spectra (OES) during PECVD process with and without copper catalyst. CH peak was matched at 431.4 nm, C_2_ peak at 516.5 nm, H peaks at 486.1 and 656.3 nm and H_2_ peaks at 608.3, 618.8, 624.6 and 628.8 nm. The relative intensity of C_2_:CH is labelled for the cases with and without a Cu foil

Based on the above experimental results, the growth mechanism of VG films on the glass substrate in the PECVD system is illustrated in Fig. 6. In the initial stage of VG growth, the hydrocarbon source gas, C_2_H_2_, is dissociated into reactive radicals and they are transported onto the glass substrate (Fig. 6a) [34]. A thin layer of amorphous carbon is believed to be formed firstly on the substrate due to the lattice mismatch between the glass and graphene (Fig. 6b) [32]. Then, the graphene nanosheets start to grow, while the amorphous carbon layer is still depositing as well, forming the carbon islands (Fig. 6c). Subsequent in-plane-oriented layer growth mode is unfavourable due to the following three mechanisms: (1) the simultaneous growth of graphene and carbon island leads to discontinuity of horizontal graphene growth; (2) because of the strain energy in the edges and defects of initial graphene, the intermediate layer may not be able to continue to form bulk crystal and thus causes a transition from 2D complete films to 3D clusters [7]; and (3) in this plasma system, the electric field is developed between bulk plasma and the surface of the substrate, and ions generated in plasma are accelerated through the sheath. The energetic ions deliver kinetic energy to the substrate by collisions on the surface, resulting in defects on the graphene film surface helping graphene growth in the vertical orientation (Fig. 6d). The VG growth is unique in plasma-enhanced chemical vapour deposition process and is not observed in typical thermal CVD processes. All these three mechanisms lead to the graphene grown as 3D clusters (Fig. 6e). In the VG growth process with copper catalyst, the hydrocarbon gas molecules are dissociated into reactive radicals on the copper surface [34] and a portion of those reactive radicals is expected to be desorbed from the copper surface and go back to the plasma. Optical emission spectra (OES) were taken during the deposition process to understand the catalytic effect of copper on radical densities as shown in Fig. 7. All the peak intensities increase significantly when the copper catalyst is employed. In particular, the intensities of C_2_ and CH peaks are quantitatively analysed because C_2_ and CH radicals have been reported as the major growing source and the terminator of graphene in plasma condition [35, 36], respectively. Thus, the relative intensity of C_2_:CH can indicate the relative contribution of the growth reaction to termination reaction. As marked in the figure, the relative intensity of C_2_:CH increases significantly from 0.69 to 1.29 after applying a Cu catalyst. Therefore, the increased reactive radicals contribute to faster formation of the VG film, resulting in the denser and faster growth of VG (Fig. 6f–g).

Chemical elements in the VG film were also analysed by EDS in order to detect the presence of copper in the VG samples. As summarized in Table 1 and Additional file 1: Figure S1, no copper element is found in the samples, indicating that the copper works as a catalyst in this PECVD process without being incorporated into the VG films.

**Table 1:** Energy-dispersive spectrometer analysis results of VG on glass grown with assistance of a copper catalyst

Element	Weight %	Atomic %
C	10.38	16.26
O	46.61	54.80
Na	6.72	5.50
Mg	2.00	1.55
Si	28.98	19.41
Ca	5.30	2.49
Totals	100.00	

**Fig. 8:**
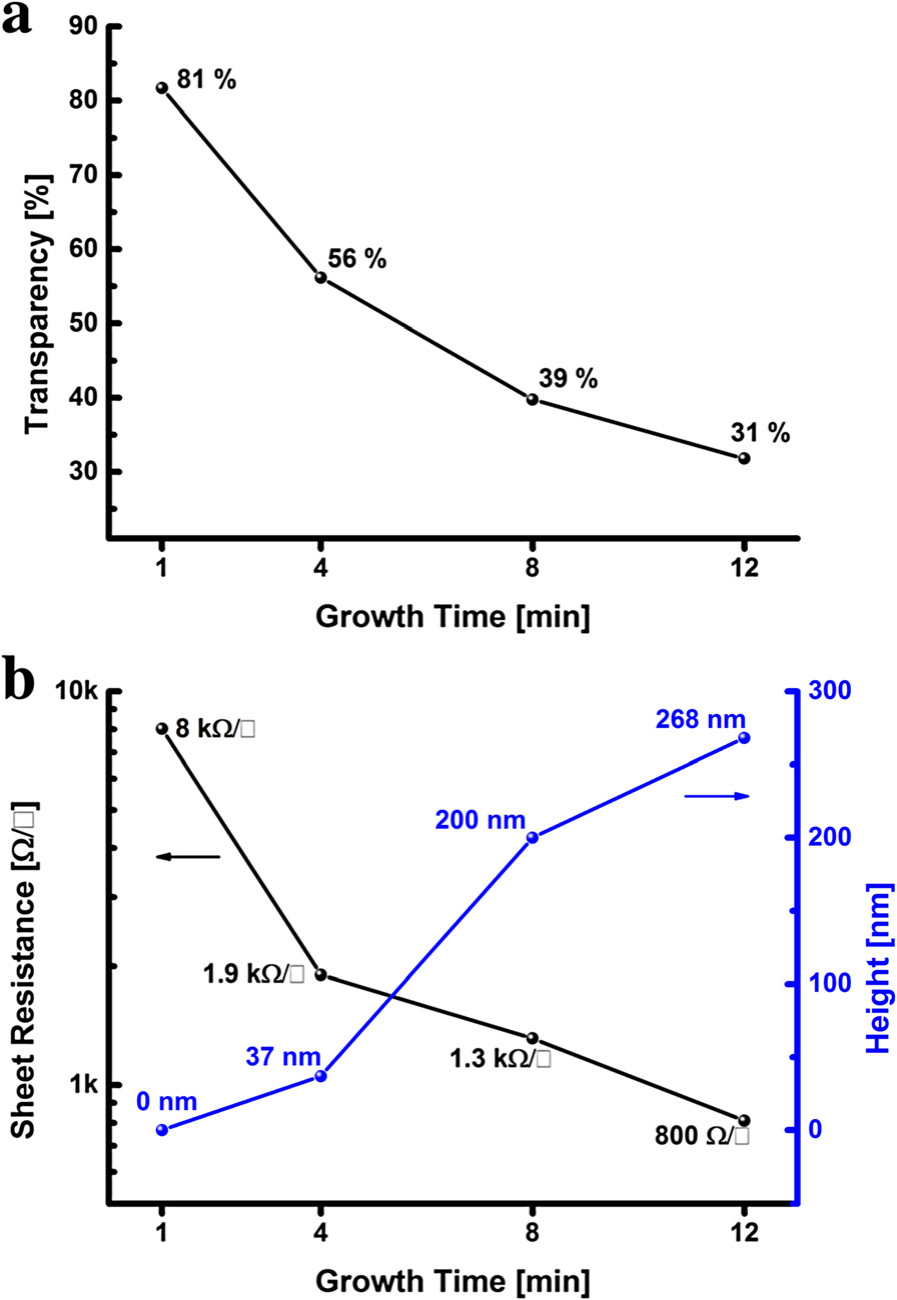
The transparency, sheet resistance and height of VG at various growth times. The transparency as a function of growth time (**a**) and the sheet resistance and VG nanosheet height as a function of growth time (**b**)

Finally, the sheet resistance, height and transparency of the VG nanosheets are plotted as functions of growth time as shown in Fig. 8. The transparency of the VG film decreases as the growth proceeds with a longer time (Fig. 8a). Meanwhile, higher VG nanosheets provide lower sheet resistance that can be attributed to the close networking of the VG nanosheets (Fig. 8b). The VG grows with the processing time, but the growth rate is not linear during the whole process. The VG shows a relatively slow growth rate of 10 nm/min in the initial stage within 4 min. As discussed earlier, the base layer is forming in the first minute and VG seeds are forming on the top of the base layer. The VG seeding process may require time and energy. Once the VG seeds are successfully formed on the base layer, the VG grows at the high growth rate of 28.8 nm/min linearly with time in the range of 4 to 12 min.

## Conclusions

In this work, vertically oriented conductive graphene film is synthesized directly on glass substrates with a copper catalyst in a low-temperature PECVD process. The catalytic mechanism of copper in the VG growth process is investigated in this work. The transparency and sheet resistance of the VG films were characterized with different growth times. The direct growth of the VG on glass substrates with copper in the PECVD process presented in this work does not require any additional substrate etching or transfer processes. This VG growth process is expected to facilitate the scaleup and makes VG production more economic for potential industrial production. The large surface area of VG films provides a big advantage in the application of electrical devices and energy storage devices.

## Additional file

Additional file 1: Figure S1. Energy-dispersive spectrometer analysis image of a VG film on glass substrate grown with assistance of a copper catalyst.
