# Supplementary material for: Copper-Assisted Direct Growth of Vertical Graphene Nanosheets on Glass Substrates by Low-Temperature Plasma-Enhanced Chemical Vapour Deposition Process
Source: Nanoscale Res Lett. 2015 Aug 4;10:308. doi: 10.1186/s11671-015-1019-8 (PMC4523503; doi:10.1186/s11671-015-1019-8)
Supplement: Additional file 1: Figure S1. — Energy-dispersive spectrometer analysis image of a VG film on glass substrate grown with assistance of a copper catalyst. [file 11671_2015_1019_MOESM1_ESM.pdf]

Additional File 1

Copper-Assisted Direct Growth of Vertical Graphene Nanosheets on Glass Substrates by Low Temperature Plasma Enhanced Chemical Vapor Deposition Process

Yifei Ma[1], Haegyu Jang[2], Changhyun Pang[1] and Heeyeop Chae[1,2,*]

[1]School of Chemical Engineering, Sungkyunkwan University (SKKU),

Suwon, 440-746, Republic of Korea

[2]SKKU Advanced Institute of Nanotechnology (SAINT), Sungkyunkwan University (SKKU),
Suwon, 440-746, Republic of Korea
E-mail: hchae@skku.edu, Tel: +82-31-290-7263.

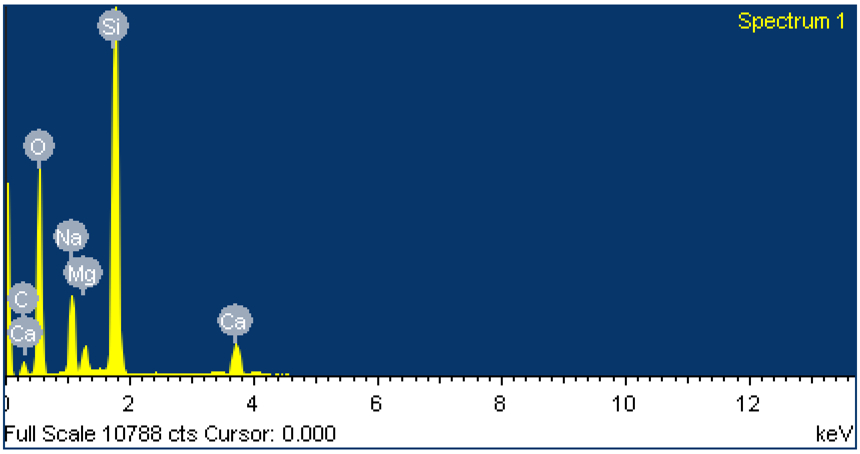

Fig. S1. Energy dispersive spectrometer analysis image of VG film on glass substrate grown with assistance of copper catalyst.
